# A singular nitric oxide synthase with a globin domain found in *Synechococcus* PCC 7335 mobilizes N from arginine to nitrate

**DOI:** 10.1038/s41598-018-30889-6

**Published:** 2018-08-21

**Authors:** Natalia Correa-Aragunde, Noelia Foresi, Fiorella Del Castello, Lorenzo Lamattina

**Affiliations:** Instituto de Investigaciones Biológicas. Facultad de Ciencias Exactas y Naturales, Universidad Nacional de Mar del Plata - CONICET, CC 1245, 7600 Mar del Plata, Argentina

## Abstract

The enzyme nitric oxide synthase (NOS) oxidizes L-arginine to NO and citrulline. In this work, we characterise the NOS from the cyanobacteria *Synechococcus* PCC 7335 (SyNOS). SyNOS possesses a canonical mammalian NOS architecture consisting of oxygenase and reductase domains. In addition, SyNOS possesses an unusual globin domain at the N-terminus. Recombinant SyNOS expressed in bacteria is active, and its activity is suppressed by the NOS inhibitor L-NAME. SyNOS allows *E. coli* to grow in minimum media containing L-arginine as the sole N source, and has a higher growth rate during N deficiency. SyNOS is expressed in *Synechococcus* PCC 7335 where NO generation is dependent on L-arginine concentration. The growth of *Synechococcus* is dramatically inhibited by L-NAME, suggesting that SyNOS is essential for this cyanobacterium. Addition of arginine in *Synechococcus* increases the phycoerythrin content, an N reservoir. The role of the novel globin domain in SyNOS is discussed as an evolutionary advantage, conferring new functional capabilities for N metabolism.

## Introduction

Nitric oxide (NO) is a free radical and a signal molecule with functional activities in all living organisms. The biological functions of NO are well documented in animals, and range from vasorelaxation, smooth muscle relaxation, platelet inhibition, neurotransmission and cytotoxicity to immunoregulation of pathophysiological processes^1–3^. Nitric oxide synthases (NOSs) are enzymes that catalyse the oxidation of the substrate L-arginine to L-citrulline and NO. Three different enzymes have been described in animals: inducible NOS (iNOS), endothelial NOS (eNOS) and neuronal NOS (nNOS). All of them present two main domains: the oxygenase containing a heme group and a tetrahydrobiopterin (BH_4_) cofactor, and the reductase domain, which provides the electrons for L-arginine oxidation. NOSs are homodimers joined by a Zn binding motif at the N-terminus^4^.

Most Gram-positive bacteria contain NOSs, though their structure differs from their animal counterparts, since they only contain the oxygenase domain. Therefore, the electron required for the catalytic activity of NOS is provided by non-specific cellular reductases^5^. An exception is a NOS from the Gram-negative bacterium *Sorangium cellulosum*, which contains a reductase module at its N-terminal end^6^. The functions of NOS in bacteria are less understood, and the actions that affect cell physiology are diverse and described less frequently. In *Bacillus*, NOS-derived NO contributes to oxidative stress resistance by activating catalases or reducing Fenton chemistry^5,7^. In *Deinococcus radiodurans*, NO regulates the recovery from UV-B radiation damage^8^. Recent results show that NOS modulates aerobic respiration and the switch to nitrate-based respiration during low-oxygen growth in pathogenic bacteria^9^. NOS also participates in the response to oxidative stress, in the biosynthesis of nitrated compounds during defence responses and in transcriptional regulation, among others^10^.

The first NOS from a photosynthetic microorganism was characterised in the microalgae *Ostreococcus tauri* (OtNOS)^11^. OtNOS is a canonical NOS with similar biochemical and spectral properties to animal NOSs. It has a high activity and seems to be independent of Ca^2+^-calmodulin, similar to animal iNOS^11,12^. Recently, new sequence similarity searches have revealed several NOS proteins in diatoms^13^ and in the green lineage, except for land plants^14,15^. Phylogenetic analysis showed that a NOS sequence from the cyanobacteria *Synechococcus* PCC 7335 (SyNOS) presents with high similarity to OtNOS^11^. Cyanobacteria are one of the most important primary producers on the Earth, and are responsible for the spread of the eukaryotic green lineage that originated from an endosymbiotic relationship between a cyanobacterial and a mitochondriate ancestor. Cyanobacteria are dominant and widespread in aquatic environments and have a high degree of morphological differentiation; their taxonomic classification is based on multi or unicellular tissue and on their types of reproduction^16,17^. The Synechococcus genus is characterised by chrococcoid cells that constitute the major component of marine picoplankton^18^. In particular, *Synechococcus* PCC 7335 is a marine free living unicellular cyanobacterium able to fix atmospheric dinitrogen^19^. In this work, we present biochemical and functional evidence of SyNOS gene structure, expression and protein activity. SyNOS presents with similar structure to canonical NOS, except for a singular N-terminal globin domain. Evidence suggests that *E. coli* cells expressing SyNOS allow bacteria to grow in minimum media containing L-arginine as the sole N source. Moreover, SyNOS-expressed bacteria have a higher growth rate in complete culture media or under N limited conditions. SyNOS is expressed and appears to be essential in *Synechococcus* PCC 7335 during exponential growth phase.

## Results

### Architecture of NOS from Synechococcus PCC 7335 (SyNOS)

**Figure 1:**
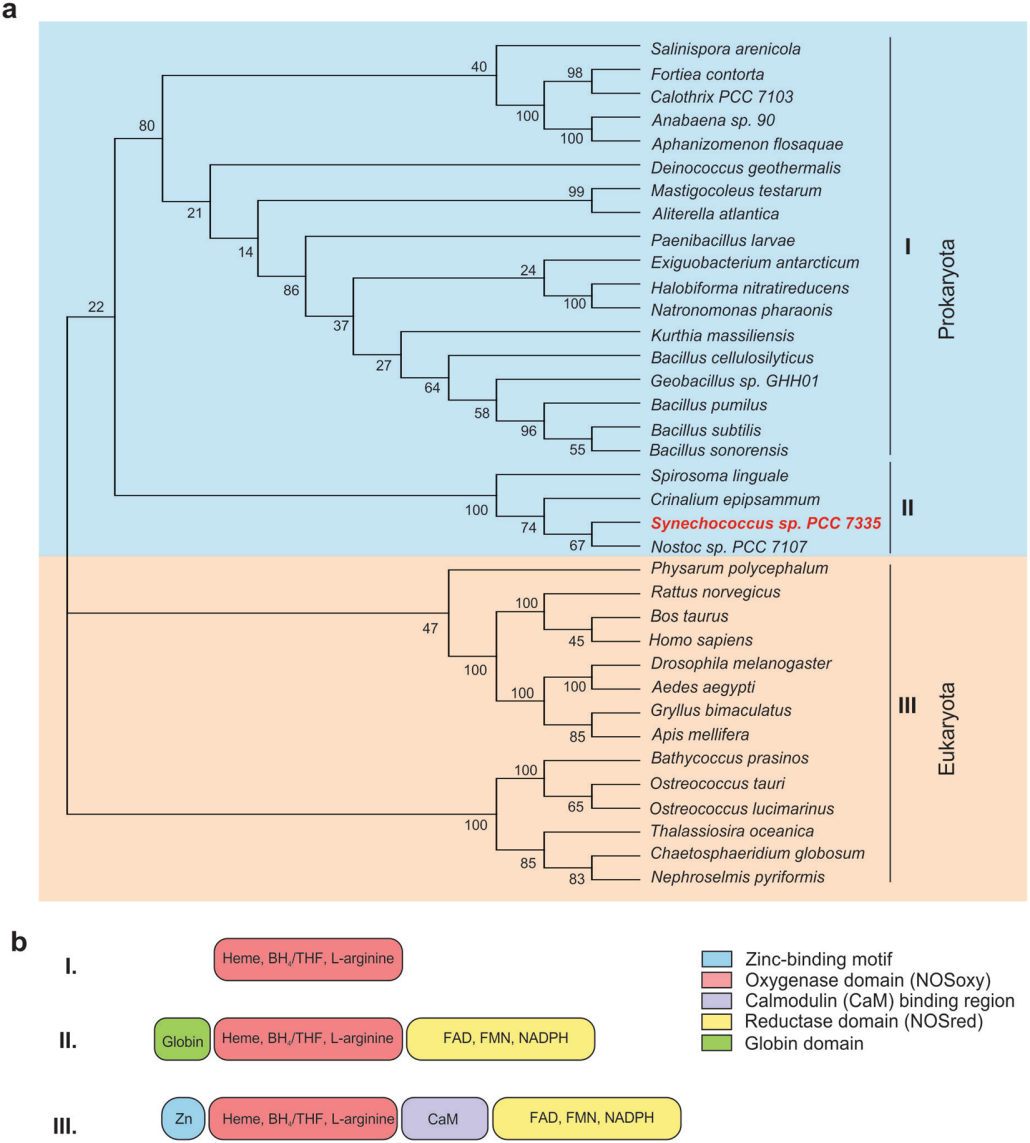
Phylogenetic tree and architecture of nitric oxide synthases (NOSs). (**a**) The amino acid sequences of the oxygenase domain of the NOS from different organisms were aligned with M-Coffee software and the alignment was evaluated with TCS software (Score 971). The relationships and distances between NOS sequences were inferred using the Maximum likelihood method based on the JTT matrix-based model. The tree with the highest log likelihood (−12544.8669) is shown. The percentage of trees in which the associated taxa clustered together is shown next to the branches. Initial tree(s) for the heuristic search were obtained automatically by applying Neighbor-Join and BioNJ algorithms to a matrix of pairwise distances estimated using a JTT model, and then selecting the topology with superior log likelihood value. Analyses were conducted using MEGA7 software. The cyanobacteria *Synechococcus* PCC 7335 is shown in red. (**b**) Representation of different NOS architectures. The oxygenase domain binds heme, the substrate L-arginine and the cofactor tetrahydrobiopterin (BH_4_) or tetrahydrofolate (THF). The reductase domain binds FAD, FMN and NADPH. In prokaryotes, NOS architectures are represented by I and II, while eukaryotes architecture is shown in III.

The analysis of the SyNOS sequence revealed that, unlike most bacterial NOSs, SyNOS has the oxygenase and reductase domains present in eukaryotic NOSs. However, the absence of the CaM-binding domain and the autoregulatory element (ACE) indicates that SyNOS activity might be independent of Ca^2+^ concentration, as occurs in all bacterial NOSs. Surprisingly, the amino acidic sequence of SyNOS showed the presence of a novel and unusual domain in its N-terminal end, which encodes a globin (Fig. 1 and Supplementary Fig. S1). Globins are heme-containing proteins involved in binding and/or transporting oxygen^20^. We searched for additional NOS sequences from photosynthetic microorganisms and performed a phylogenetic analysis of NOS oxygenase domains (Fig. 1a). The phylogenetic tree displays two main branches that cluster eukaryotic and prokaryotic NOSs. In the prokaryotic group, the cyanobacterial NOS is only present in the orders Nostocales (*Fortiea contorta*, *Calothrix PCC 7103*, *Anabaena* sp. 70, *Aphanizomenon flosaquae*, *Mastigocoleus testarum* and *Nostoc* PCC 7107), Oscillatorales (*Crinalium eppipsamum*), Chroococcidiopsidales (*Aliterella atlantica*), and Synechococcales (*Synechococcus* PCC 7335). Cyanobacterial NOS oxygenase can be divided into two clusters (I and II) with two different architectures (Fig. 1a,b). One branch (I) has the typical bacterial NOS structure with only the oxygenase domain. The second branch is the cyanobacterial branch (II) corresponding to species that have a complete NOS with a globin domain, with the exception of the bacteria *Spirosoma linguale* (Fig. 1a,b). NOS sequences from eukaryotic photosynthetic organisms contain both the oxygenase and reductase domains, connected by a sequence homologous to the CaM-binding motif, and clusters in a separated branch respect to non-photosynthetic organisms.

**Figure 2:**
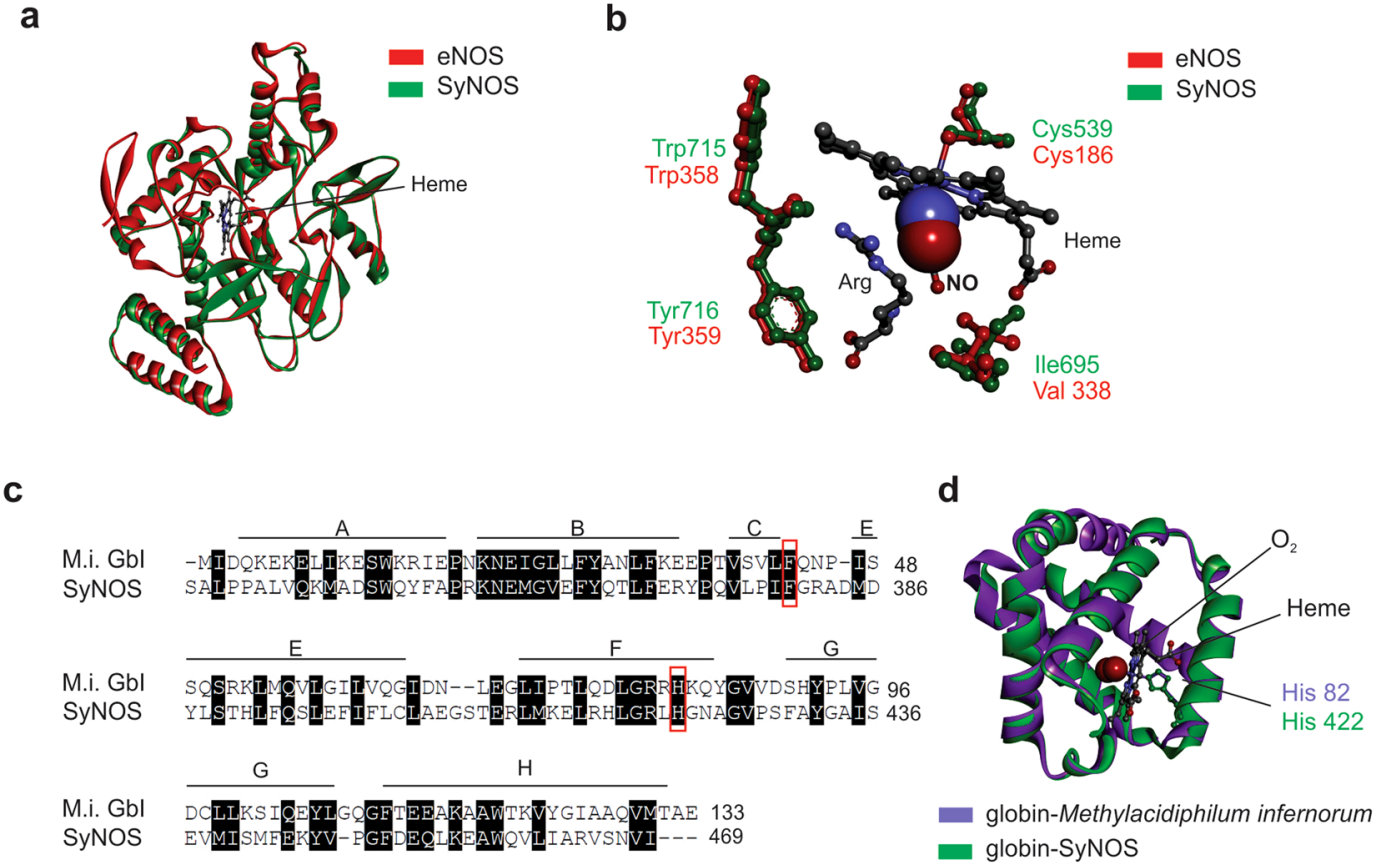
Nitric oxide synthase (NOS) from *Synechococcus* PCC 7335 (SyNOS) conserves the tridimensional structure of canonical NOS together an active globin domain at its 5′terminus. (**a**) Ribbon diagram of SyNOS oxygenase domain (green) superposed to bovine endothelial NOS (eNOS, PDB code 1d1p, red). (**b**) Conservation of key amino acid residues in the active sites of eNOS (binding of arginine and release of NO), except that Val338 in eNOS is replaced by Ile695 in SyNOS. (**c**) Alignment of SyNOS globin domain with *Methylacidiphilum infernorum* globin (M.i. Glb). Alpha helices from A to H are shown. D helix is absent in prokaryotic globins. The invariant residues Phe380 (named CD1) and His422 (named F8) are boxed in red. Black boxes indicate conserved residues between sequences. (**d**) Ribbon diagram of the extra N-terminal globin domain of SyNOS superposed to *M. infernorum* globin I (PDB code 3s1i) highlighting the heme position together with the binding site for O_2_ and the coordinating His residue.

Structural models based on best Protein Data Bank (PDB) templates were built for SyNOS oxygenase (SyNOSoxy). Fig. 2a shows the precise superposition between the predicted three-dimensional structures of the SyNOSoxy domain superposed on human eNOSoxy (PDB code 4D1O). In SyNOSoxy, the heme group is stabilised by binding to the sulphur atom of Cys539 (Fig. 2b). The residues that interact with the substrate L-arginine (L-Arg) (Trp715 and Tyr716) are fully conserved in the SyNOSoxy sequence. The structural difference in SyNOS, with respect to eNOS, is a change from Val338 (eNOS) to Ile698 (SyNOS) (Fig. 2b). The presence of Ile instead of Val near the heme pocket is observed in all bacterial NOSs, and determines their characteristic kinetic profile^21,22^. In the iNOS isoform, the single mutation from Val to Ile slows down NO synthesis and NO release^23^. The SyNOS globin domain was aligned and modelled based on the coordinates of the globin from *Methylacidiphilum infernorum* (PDB code 3S1I)^24^ (Fig. 2c). Globins occur in all living organisms and can be classified into single-domain globins and chimeric globins. The latter comprises flavohaemoglobins with a C-terminal FAD-binding domain and globin-coupled sensors^20^. The SyNOS globin domain corresponds to a classical bacterial globin composed of six helices (from A to H), lacking the D-helix. The hallmark residues of the globin structure Phe (CD1) and His (F8)^25^ are conserved in SyNOS. Figure 2d shows the superposition of *M. infernorum* globin structure with SyNOS globin domain. These results suggest that SyNOS has two heme groups, one in the oxygenase domain and the other in the globin domain.

### Recombinant expression of SyNOS in bacteria

**Figure 3:**
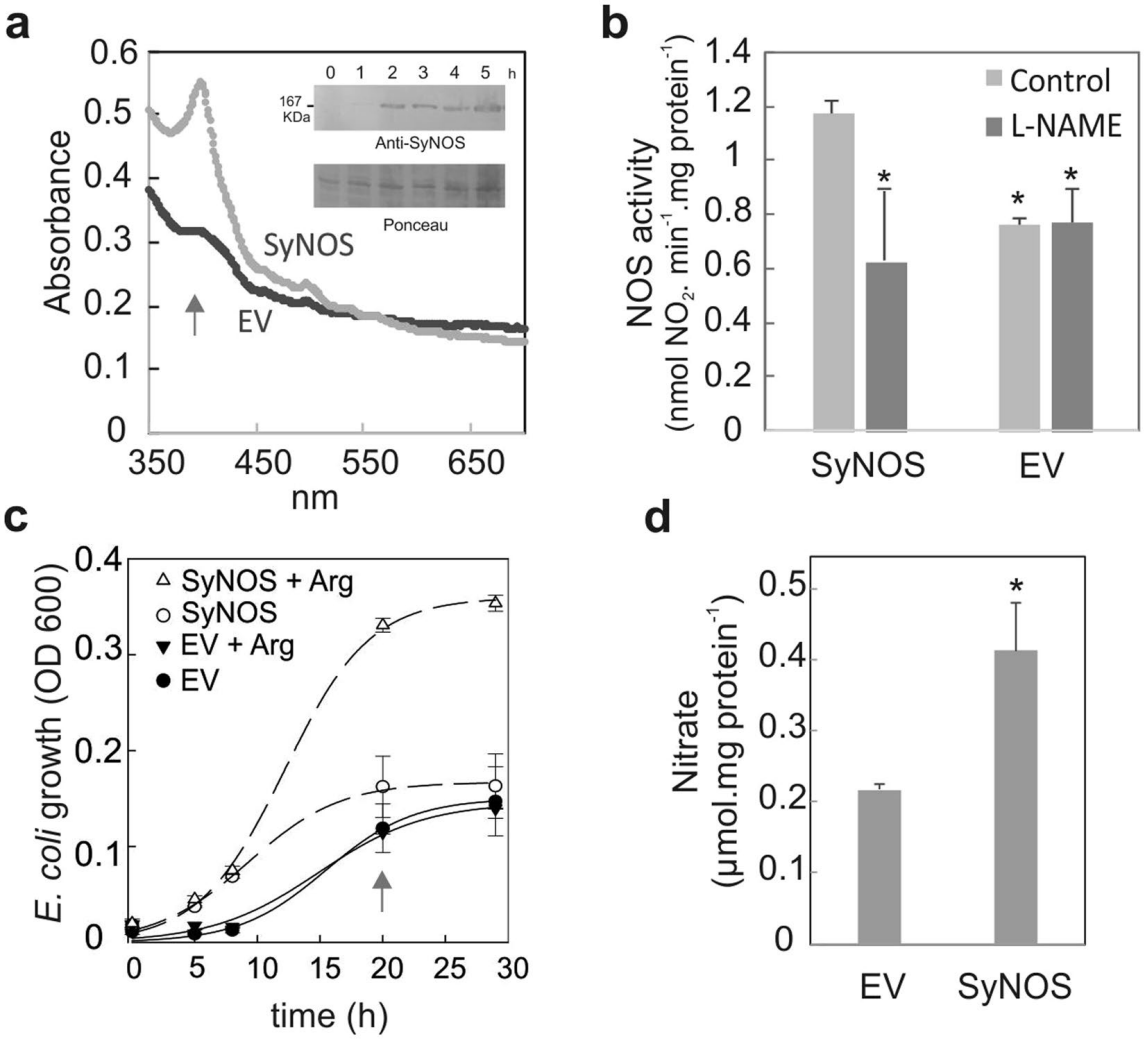
Expression and activity of recombinant SyNOS allows *E. coli* cultures grow with arginine as N source. The open reading frame of SyNOS was cloned into pET15b or pET24a vectors with a c-myc tag in the C terminus. The expression of SyNOS was induced by addition of 0.1 mM IPTG. (**a**) Wavelength spectra of *E. coli* culture extracts grown in LB medium expressing pET15b-SyNOS or the empty vector pET15b (EV). The arrow indicates the peak at 396 nm of the SyNOS protein containing a high spin heme prosthetic group. Inset: Immunoblot showing the expression of SyNOS revealed with anti-myc antibody. Full length blots are presented in Supplementary Fig. S2. (**b**) NOS activity in extracts of *E. coli* grown in LB medium expressing SyNOS or the empty vector pET15b (EV), with or without the addition of 5 mM L-nitroarginine methyl ester (L-NAME). Error bars represent ± SE (n = 3). Asterisks indicate significant differences compared to SyNOS without the inhibitor (t-test, p < 0.05). (**c**) Curves of *E. coli* cultures growing in media deficient of N or media containing arginine as the only source of N. Bacteria expressing SyNOS or the empty vector pET24a (EV) (OD 600 ~ 1) were diluted (1/100) in minimum media containing 0.2% (w/v) glucose with or without 0.2% (w/v) arginine (N source). Culture growth was quantified by OD 600. Error bars represent ± SE (n = 3). (**d**) Nitrate content in cultures grown for 20 h in minimum media containing arginine (arrow in panel **c**). Error bars represent ± SE (n = 3). The asterisk indicates a significant difference compared to EV (t-test, p < 0.05).

The full length of SyNOS was cloned with a c-myc tag in the bacterial expression vectors pET24a and pET15b. The expression of SyNOS was detected after two hours of induction with isopropyl β-D-1-thiogalactopyranoside (IPTG) in *E. coli* (Fig. 3a, inset). Extracts from *E. coli* that express SyNOS presented a peak at 396 nm, indicating that the NOS protein contained a five-coordinate high-spin heme iron (Fig. 3a). NOS activity was detected in SyNOS-expressed cell extracts (Fig. 3b). Treatment with the specific NOS inhibitor L-NG-nitro arginine methyl ester (L-NAME) diminished nitrite production. Even though a basal nitrite-producing activity was detected in bacterial extracts expressing the empty vector (EV), it was not inhibited by L-NAME, and seems not to be SyNOS-dependent (Fig. 3b). We analysed the growth of bacteria expressing SyNOS in minimum medium containing glucose (0.2%, w/v) as the C source, with or without arginine (0.2%, w/v) as the sole N source. Figure 3c shows that the growth of bacteria expressing SyNOS or EV was severely affected in media without N. However, when arginine was used as the sole N source, the expression of SyNOS allowed bacteria to triplicate their growth yield, compared to EV. These results indicate that SyNOS is functional in *E. coli*, providing the advantage of growth in medium containing arginine as the sole N source. After 20 hours of culture, SyNOS-expressing bacteria had a two-fold higher NO_3_^−^ content, in comparison to EV (Fig. 3d). This result suggests that the NO produced by SyNOS, from arginine, is oxidized to NO_3_^−^.

**Figure 4:**
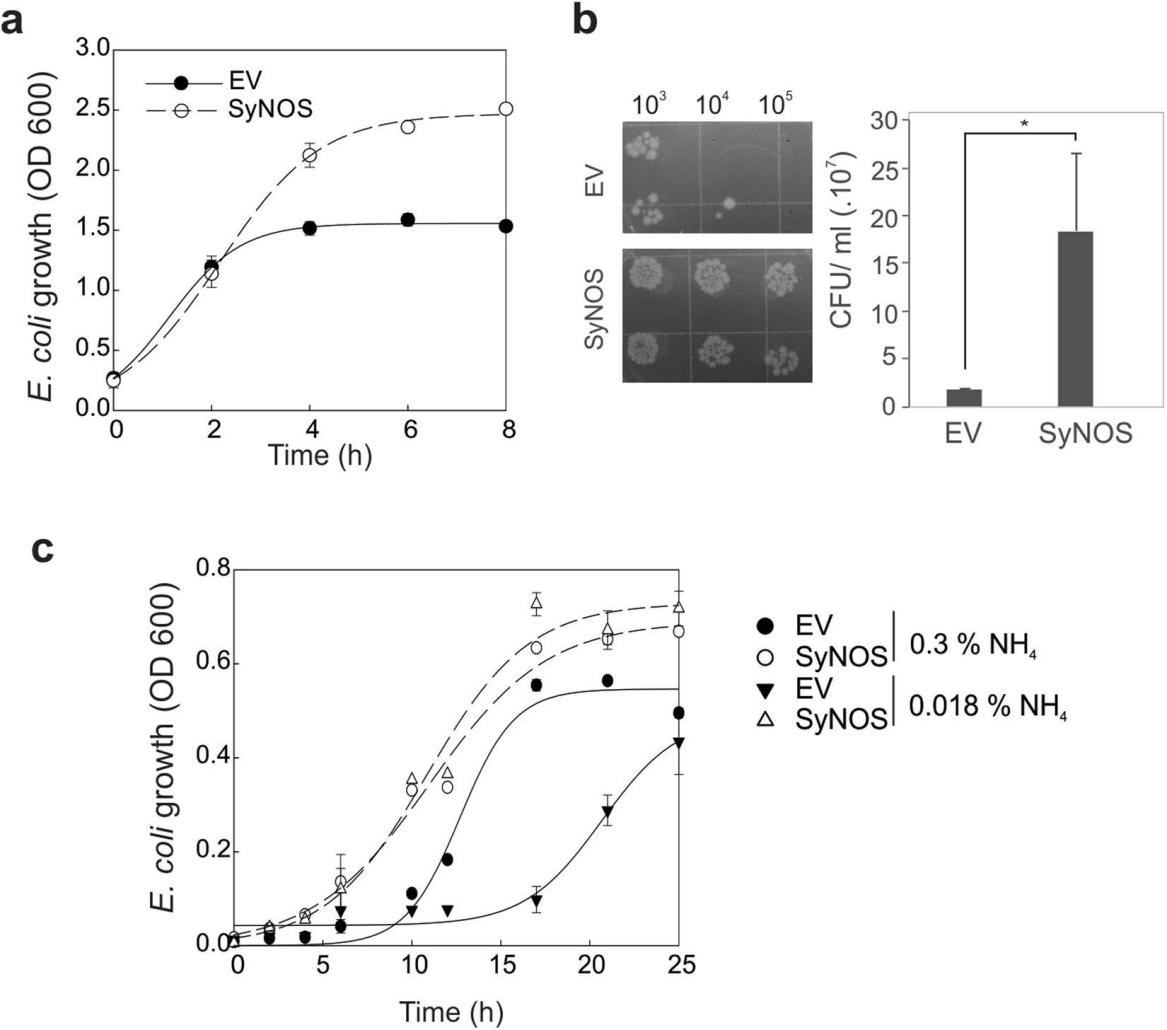
Expression of recombinant SyNOS increases the maximum OD of *E. coli* culture growing in either sufficient or deficient N conditions. (**a**) *E. coli* cultures expressing recombinant SyNOS containing a myc-tag or the empty vector pET24a (EV) were grown in LB media and induced with 0.1 mM IPTG. Growth was followed by measuring OD 600. Error bars represent ± SE (n = 4). (**b**) Dilutions (10^−3^, 10^−4^ and 10^−5^) of *E. coli* cultures expressing SyNOS or EV and induced with IPTG 0.1 mM for 4 h were plated in LB agar and grown for 18 h at 37 °C. The number of colony-forming units (CFU) per ml from *E. coli* was quantified. Error bars represent ± SE (n = 3). The asterisk indicates a significant difference compared to EV (t-test, p < 0.05). (**c**) Curves of *E. coli* cultures growing in media with sufficient or deficient N conditions. Bacteria expressing SyNOS or EV (OD 600 ~ 1) grown in LB were diluted (1/100) in minimum media containing 0.2% (w/v) glucose with 0.3% or 0.018% (w/v) NH_4_Cl (N source). Culture growth was quantified by OD 600. Error bars represent ± SE (n = 3).

The cultures expressing SyNOS, growing in Luria Broth (LB) media, reached a 65% higher OD 600 at the stationary phase, compared to bacteria expressing the EV (Fig. 4a). The higher OD corresponds to an augmented cell population, according to the colony forming units (CFU) counted on LB agar (Fig. 4b). In addition, when the cell viability was evaluated using the probe Sytox Green, the result indicated that the culture expressing SyNOS had increased viability (Supplementary Fig. 3). The increased growth reached at stationary phase in the BL21 strain was reproduced in the wild type *E. coli K12*-derived strain, *RP437*, expressing SyNOS under the control of a salicylic acid-induced expression vector (Supplementary Fig. 4). To analyse the growth of bacteria under N deficient conditions, *E. coli* expressing SyNOS or EV were inoculated in minimum medium containing glucose (0.2%, w/v) as the C source with 0.3% (w/v) NH_4_Cl (sufficient N condition) or 0.018% (w/v) NH_4_Cl (deficient N condition). *E. coli* expressing SyNOS or EV under the sufficient N condition grew at a similar rate (Fig. 4c). However, under the limited N condition, SyNOS-expressing bacterial cells showed a higher growth rate compared to EV, suggesting that SyNOS confers tolerance to N deficient conditions.

### Expression of SyNOS in Synechococcus PCC 7335: Relationship with N metabolism and cyanobacterial growth

**Figure 5:**
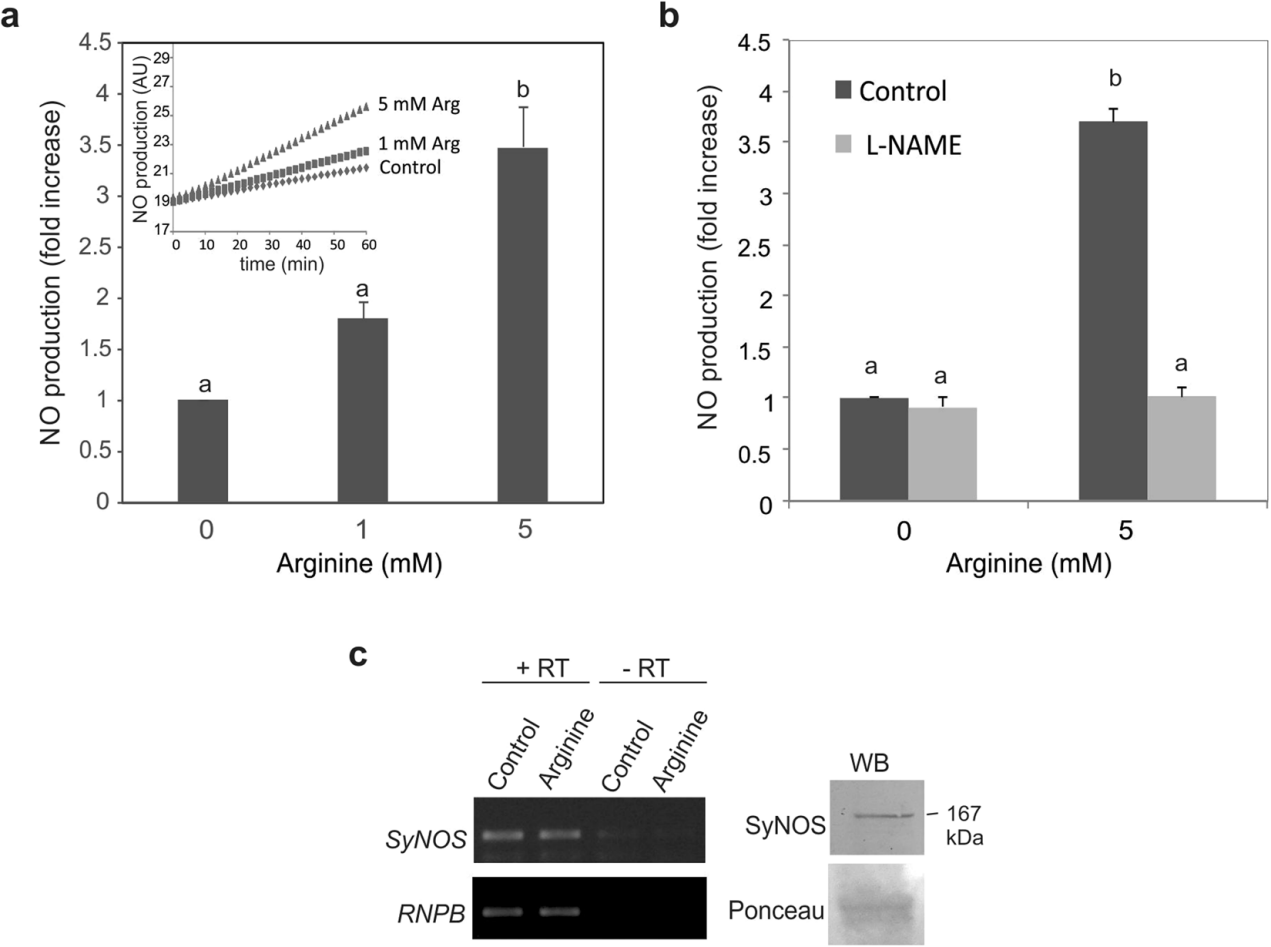
Arginine increases NO production in *Synechococcus* PCC 7335. (**a**) NO production by Synechococcus grown in ASNIII medium (OD 600 = 0.3) with or without the addition of different concentrations of arginine. NO was detected with the fluorescent probe DAF-FM DA using a microwell fluorometer over a 60 min period. Data are expressed as fold increase with respect to the control. Error bars represent ± SE (n = 3). Letters denote significant differences among treatments (ANOVA test, F_2,6_ = 26.512; post hoc test Holm-Sidak method, p < 0.05). Inset: NO fluorescence expressed as arbitrary units (AU). A representative graph of three independent experiments is shown. (**b**) NO production in *Synechococcus* PCC 7335 grown in ASNIII medium treated with 5 mM arginine in the presence or absence of 5 mM of L-NAME. Error bars represent ± SE (n = 4). Letters denote significant differences among treatments (ANOVA test, F_3,8 _ = 230.535; post hoc test Holm-Sidak method, p < 0.001). (**c**) RT-PCR and western blot (WB) analysis showing the expression of SyNOS in *Synechococcus* PCC 7335. The RNase P RNA (RNPB) was used as a control for cDNA loading. SyNOS was detected with a specific antibody (Genscript). Full length blots are shown in Supplementary Fig. S5.

NO production in *Synechococcus* PCC 7335 was measured in the presence of different amounts of L-arginine, using the specific NO probe DAF-FM DA. Fig. 5a shows that the addition of L-arginine increases NO production in a dose-dependent manner. NO production could be completely inhibited by the specific NOS inhibitor L-NAME (Fig. 5b), suggesting that the NOS enzyme is responsible for NO production. The expression of SyNOS was analysed in *Synechococcus* PCC 7335 by RT-PCR and immunoblot. Figure 5c shows that *SyNOS* transcript is expressed in actively growing cyanobacteria. The addition of L-arginine to the culture did not increase *SyNOS* transcript levels, indicating that there is sufficient NOS enzyme to metabolize the substrate arginine added to the culture (Fig. 5c). Figure 5c (right) shows the immunoblot revealing the SyNOS protein with an estimated size of 167 kDa. Arginine-dependent NO production was dramatically diminished in iron (Fe) deficient media, suggesting that Fe is essential for SyNOS activity (Supplementary Fig. S6).

**Figure 6:**
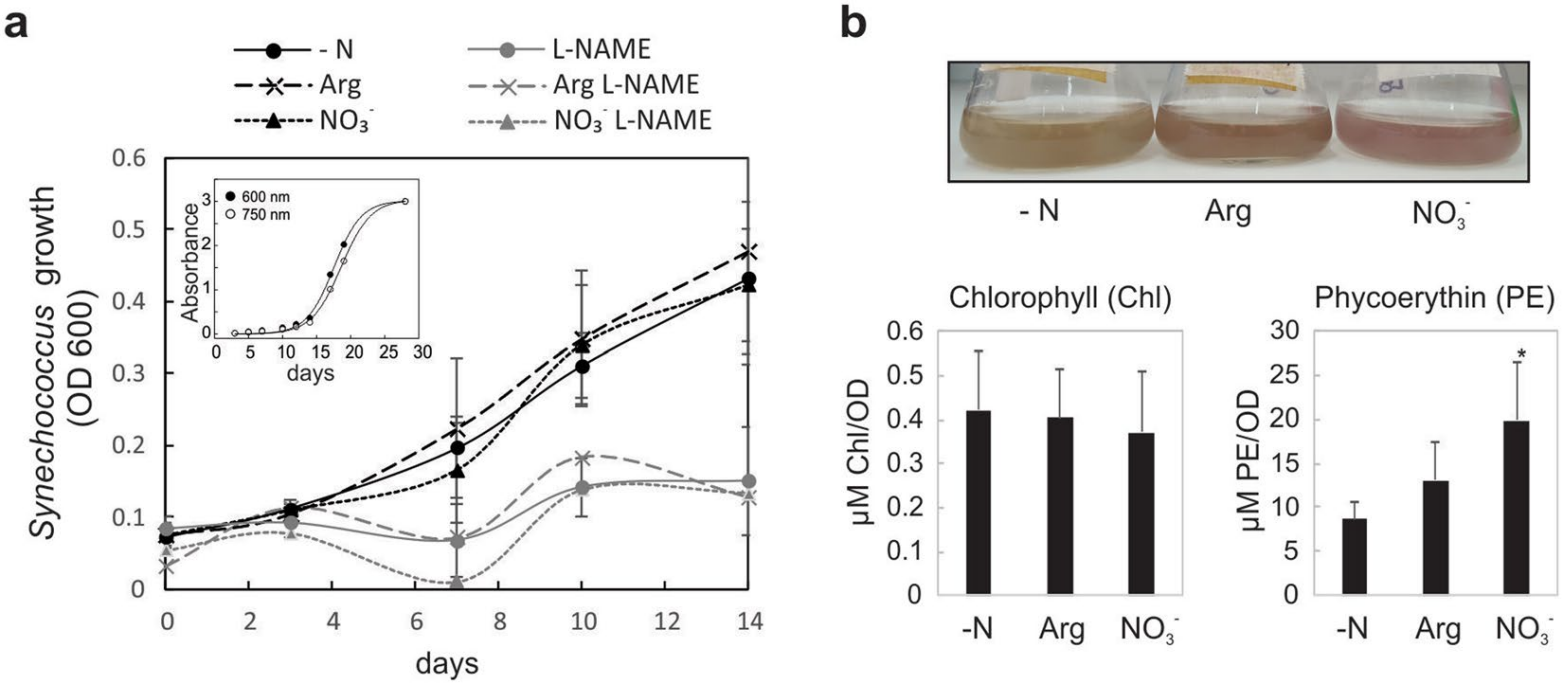
Growth and pigment contents in *Synechococcus* PCC 7335. (**a**) *Synechococcus* PCC 7335 was grown in ASN III medium without N (−N) or with the addition of 1 mM arginine (Arg) or 1 mM NO_3_^−^, and with or without 1 mM L-NAME. Culture growth was measured by OD 600. Inset: growth curve to stationary phase of *Synechococcus* PCC 7335 growing in ASNIII medium followed by absorbance at 600 and 750 nm. Error bars represent ± SE (n = 3). (**b**) Chlorophyll (Chl) and phycoerythrin (PE) content in Synechococcus cultures grown 15 days in ASN III medium without N (−N) or with the addition of 1 mM Arg or 1 mM NO_3_^−^. Photographs of the cultures (top) and quantification of pigments (bottom) are shown. Error bars represent ± SE (n = 3). The asterisk indicates significant difference as compared to −N. (Man-Whitney test, p < 0.05).

*Synechoccocus* PCC 7335 is an N-fixing cyanobacteria; thus, it is able to grow in medium without N (Fig. 6a). Therefore, we analysed whether the addition of arginine affects *Synechococcus* growth. Cyanobacterial growth curves were evaluated in ASNII media without N supplemented, with 1 mM arginine or NO_3_^−^. Figure 6a shows that neither arginine nor NO_3_^−^ modified cyanobacterial growth. However, the addition of L-NAME severely affected normal *Synechococcus* growth, independent of the N source (Fig. 6a). Moreover, different colours were observed in the cultures without N, with arginine and with NO_3_^−^ cultures, after 15 days of growth (Fig. 6b). We analysed the level of chlorophyll (Chl) and phycoerythrin (PE) pigments. While no differences were found in Chl content, nitrate supplemented cultures showed significantly higher PE compared with cultures with no N, and intermediate amounts of PE were observed with arginine as the N source (Fig. 6b). Wyman *et al*.^26^ showed that *Synechococcus WH7803* cells accumulate PE when cultivated in N-sufficient media, and act as a reservoir for N that maintains productivity when the external availability of N is interrupted. Figure 6b supports a relationship between the form of available N, the concentration of PE and the growth rate of *Synechococcus* at the initial time of cultivation.

## Discussion

NOSs are oxidoreductase enzymes found in all living organisms. Intensive research has been carried out to investigate the presence of NOS in photosynthetic organisms. The first characterized NOS in a photosynthetic species was from the green algae *Ostreococcus tauri*^11^. Since that report, further bioinformatic studies allowed the discovery of many NOSs in other photosynthetic organisms. A search for the presence of algal NOSs in the onekp database, and in available genomes, led to the identification of 15 NOS-like sequences in a total of 265 algal species, while no NOSs were found in land plants^15^. This analysis showed that, in spite of a global congruence in the evolutionary history of NOS proteins, NOS sequences in photosynthetic microorganisms are circumscribed to specific orders or families.

The NOS from *Synechococcus* PCC 7335 (SyNOS) contains an unusual globin domain also found in diatoms from the *Pseudo-nitzschia*, *Thalassiosira* and *Skeletonema* genera^13^. Transcriptomic studies have indicated that two different transcripts are found in centric diatom species, one of them showing the globin domain, and the other one lacking this domain^13^. The functional relevance of this dimorphism is yet to be elucidated. The presence of a globin domain and a reductase domain in the same molecule resembles bacterial flavohaemoglobins^25,27^. Flavohaemoglobins are widely expressed among bacteria and yeasts and are involved in physiological roles associated with cell responses to oxidative/nitrosative stresses. Bacterial globins are known to interact with NO and either convert it aerobically into nitrate or anaerobically into nitrous oxide^28^. Our results show, for the first time, that *E. coli* expressing SyNOS can grow in a medium containing arginine as the sole N source, indicating that SyNOS possesses the ability to metabolise and assimilate the N available as arginine. Furthermore, higher levels of free NO_3_^−^ were detected in SyNOS-expressing cultures. This result is consistent with the idea that the NOS activity in recombinant *E. coli* is responsible for the growth in the medium containing arginine as the N source. Moreover, the expression of SyNOS was also associated with higher bacteria growth yield and increased growth rate under N deficiency. If the NOS reductase domain of SyNOS could transfer e- to the NOS, the globin domain could behave as a flavohaemoglobin taking the NO generated by the oxygenase domain and producing NO_3_^−^ in an aerobic environment. This NO_3_^−^ may re-enter into the cell N metabolism to generate new amino acids via nitrate reductase (NR) and nitrite reductase (NiR) activities. This is not merely a futile N-cycle, but an indication of a greater metabolic plasticity to use other N sources and mobilise them to different forms required by the cell. Arginine constitutes between 5–7% mol of the total free amino acids in sea water^29,30^, and transporters in cyanobacteria that could be functional for internalizing arginine have been described^31,32^. Moreover, the NOS globin domain might serve to detoxify excess NO and thereby down-regulate/modulate SyNOS activity. In this study, we were not able to express SyNOS without the globin domain in *E. coli*, suggesting that folding and/or protein stability is affected in the truncated protein.

Experiments performed with *Bacillus subtilis* wt and Δ*nos* have suggested that NOS activity is required for the maintenance of normal bacterial growth at the log-to-stationary transition phase^5^. A more recent study indicated that the NOS of *Staphylococcus aureus* acts in concert with a flavohaemoprotein to form NO_3_^−^, in order to maintain membrane bioenergetics and colonization^33^. Under microaerobic conditions, the NO derived from NOS activity inhibits the cytochrome oxidases in *Staphylococcus aureus*, thereby conducting electrons to the periplasmic NR complex, which reduces NO_3_^−^ to NO_2_^−^ and maintains the membrane potential^33^. In Chlamydomonas, electrons from NAD(P)H in the diaphorase domain of NR are transferred to the truncated haemoglobin THB1, which scavenges NO through its dioxygenase activity, producing NO_3_^−^^34,35^.

The change of the Val residue found in animal NOS to Ile in the heme pocket of SyNOS appears not to be neutral. Wang *et al*.^23^ characterised the V346I mutant of iNOS and found that the mutant exhibited lower NO synthesis with uncoupled NADPH consumption. V346I iNOS has enhanced NADPH-dependent NO dioxygenase activity^23^. In the case of SyNOS, the oxygenase domain of SyNOS, with Ile instead of Val, may retain NO within the catalytic site to be oxidised by the globin domain.

Several studies indicate that the NO generated by marine phytoplankton is involved in growth, physiology and cell death^11,36–40^. Although many species of phytoplankton do not have a NOS, it has been proposed that NO comes from NR activity and/or from the mitochondrial electron transport chain^41–43^. Regardless of its origin, in marine microorganisms, NO is involved in metabolism regulation and signalling, and in defense against metabolic and oxidative stresses. Treatment with a NO donor protects the growth and pigment content and enhances antioxidant activities in cyanobacteria exposed to UV-B^44^. Moreover, the growth of microalgae caused by UV, heavy metals and pesticides was shown to be alleviated by exogenous NO^45^. The protective effect of NO seems to be due to its ability to detoxify ROS, mainly in the photosynthetic apparatus^46^. Taken together, there is now strong evidence to suggest that NO is a novel factor regulating growth in phytoplankton.

Some studies in higher plants have shown that NO is a potential N source, given that a positive effect on plant growth, amino acid and protein content was observed when these plants were fumigated with NO or NO_2_^47–49^. Overexpression of non-symbiotic haemoglobin 1 or 2 (*GLB1* and *GLB2*) genes in Arabidopsis allows plants to use atmospheric NO as a source of N^50^. Authors have proposed that NO is oxidised enzymatically to NO_3_^−^ by GLBs, and then can be reduced to ammonia by NR and NiR, to finally be incorporated into amino acids^50^. Frungillo *et al*.^51^ also linked NO with N metabolism and assimilation. Nitrosothiol signalling modulates N assimilation pathways by suppressing NO_3_^−^ uptake, through transporters and the activity of NR. Our results indicate the presence of a NOS enzyme that harbours a globin domain, in a marine cyanobacterium. SyNOS possesses, in just one molecule that behaves like a complex, all the components required to generate NO_3_^−^ from arginine. This singular NOS may have profound implications for devising strategies to improve the efficiency of use of N in crop plants, which could lead to a decrease in fertilizer overload in cultivated soils.

## Materials and Methods

### Materials

L-arginine, tetrahydrobiopterin (BH_4_), sulfanilamide (SA), N-naphthyl-ethylenediamine (NED), c-Myc agarose column and c-Myc antibody were all purchased from Sigma-Aldrich. The NOS inhibitor L-NAME was obtained from Calbiochem (San Diego, CA). The polyclonal anti-SyNOS was acquired from Genscript (USA). Restriction enzymes, 4-amino-5-methylamino-2′,7′-difluorofluorescein diacetate (DAF-FM DA), Bacto-yeast extract, IPTG and *Escherichia coli* DH5α cells were purchased from Invitrogen (USA). BL21 (DE3) pLys cells, and both pET24b and pET15b were obtained from Novagen (Madison, WI). *Synechococcus* PCC 7335 was purchased as part of the Pasteur Culture Collection (PCC, Paris, France).

### Phylogenetic analysis and homology modelling

Protein sequences with similarity to SyNOS were retrieved using BLAST using the nonredundant database from GenBank, HMMER^52^ using EMBL genome databases and the onekp database^15^. The NOS oxygenase domains were extracted and multiple sequence alignment was performed using M-Coffee^53^ with default parameters. The alignment was assessed with TCS^54^ software prior phylogenetic reconstruction. Maximum likelihood phylogenetic reconstruction was performed with Mega7^55^. Bootstrapping (100 replicates) was used to assess tree branching support. The full length of SyNOS and human NOS were aligned using ClustalX software^56^ and colored the conservative amino acid with GeneDoc. Supplementary Table S1 shows the accession number of NOS sequences used for phylogenetic analysis.

Structural models were built with MODELER v 9.5^57^ using ClustalX and FFAS03-derived alignments. The template corresponds to the crystal structure of the NOS oxygenase region of *Bos taurus* eNOS. Structural superposition of SyNOS, *B. taurus* eNOS (PDB code 1d1p) and globin from *Methylacidiphilum infernorum* (PDB code 3s1i) was performed with the program SuperPose^58^. The figures were drawn using Discovery Studio Visualizer 1.6 software (Accelrys).

### DNA manipulation and *E. coli* culture growth

DNA from *Synechococcus* PCC 7335 was extracted according to Cai and Wolk^59^ with minor modifications. Cells of *Synechococcus* PCC 7335 in the mid-log phase of growth were harvested from 50 ml of liquid culture and suspended 400 µl in 10 mM Tris-0.1 mM EDTA, pH 8. Cell lysis was performed with 100 µl of glass beads 1% SDS and 400 µl of phenol: chloroform (1:1, vol/vol). The mixture was subjected to a cycle of vigorous vortexing for 1 min followed by cooling on ice for 1 min for a total of four times and centrifuged 12000 g for 10 min. The aqueous phase was transferred to other tube and treated with RNAase for 30 min at 37 °C. Half vol of phenol and chloroform (1:1, vol/vol) was added and separated by centrifugation at 12000 g for 5 min. DNA was precipitated from aqueous phase by adding 1/10 vol of sodium acetate 3 M pH 5.2 and 2.5 vol of cold ethanol 100%. DNA was washed twice with ethanol 70% and 100% and resuspended in sterile water.

The SyNOS coding sequence was amplified from DNA extracted from *Synechococcus* PCC 7335 and cloned into pET24a or pET15b c-myc tag in the C-terminal. The product was purified and cloned into pET24a (Novagen) with *EcoRI* and *XhoI* restriction enzymes, and into the pET15b vector with *NdeI* and *BamHI* restriction enzymes. The vectors pET24a-SyNOS and pET15b-SyNOS were sequenced to verify each clone. Vectors were used to transform BL21 protease-deficient chemically competent *E. coli*. Protein expression was induced at OD 600 = 0.3 by the addition of 0.1 mM IPTG for 1.5 h. The heme precursor 8-aminolevulinic acid (500 μM) and arginine (1 mM) were added at the moment of induction. Aliquots of cultures were diluted (1/100) to minimal media containing 5.44 g KH_2_PO_4_ and 6 ml salt solution (10 g MgSO4.7H_2_O, 1.0 g MnCl_2_.4H_2_O, 0.4 g FeSO_4_.7H_2_O and 0.1 g CaCl_2_.2H_2_O per l) in 1 l of distilled water^60^. Glucose (0.2%, w/v) served as the carbon source with or without the amino acid arginine (0.2%, w/v), or NH_4_Cl (0.3% and 0.018%, w/v) as the nitrogen source. The growth was followed by measurement of OD 600.

### NOS activity and nitrate content

Recombinant protein expression was induced as indicated above. Cells from 200 ml LB culture were harvested after 5 h of induction, and resuspended in 2 mL of buffer (100 mM Tris-HCl, pH 7.4, NaCl 50 mM, 1 mM L-Arg, 100 μM BH_4_, 10% [v/v] glycerol, 1 mM PMSF) and lysed by pulsed sonication (six cycles of 30 s). Cell debris was removed by centrifugation, and the supernatant was used for determination of NOS activity and spectra. Bacteria expressing the empty vector were used for control experiments. NOS activity was assayed as described by Wang *et al*.^61^ with minor modifications. Ten microliters of protein extracts were incubated in 50 μl final volume containing 20 μM H_4_B, 3 mM arginine, 0.3 mM DTT, 0.1 mg/ml BSA, 10 U/ml SOD, 346 U/ml catalase, 4 μM FMN and FAD, and 10 μl of enzyme extract was added into a 96-well microplate and incubated at 4 °C for 30 min. The reaction was initiated by adding 1 mM NADPH. After 30 min at 37 °C, 1.5 mM of the NOS inhibitor S-ethylisotiourea (SEITU) was added to stop the reaction. Excess NADPH was consumed by the addition of 10 U/ml lactate dehydrogenase (LDH) and 10 mM sodium pyruvate. Nitrite was detected by measuring the absorbance at 540 nm using a microplate reader, by the Griess method^62^.

Nitrate was quantified by the method described by Cataldo *et al*.^63^. Cell pellets were resuspended in PBS buffer and lysed by pulsed sonication (six cycles of 30 s). Cell debris was removed by centrifugation. Nitrite content in samples was measured by the Griess reaction^62^. Briefly, 50 μl of samples were incubated with 50 μl SA for 10 min at room temperature. Then, 50 μl of NED was added to the sample and incubated for 10 min. The absorbance was measured at 540 nm. Nitrate plus nitrite was quantified by the nitration of salicylic acid^63^. Samples (10 μl) were incubated with 40 μl of salicylic acid (5 mg/ml in H_2_SO_4_) for 20 min. The samples were neutralised by the addition of 950 μl of NaOH 2 N, and the absorbance at 410 nm was determined. Nitrate content was determined by the subtraction of nitrate and nitrite content.

### RT-PCR analysis

RNA from *Synechococcus* PCC 7335 was extracted with TRizol (Invitrogen). The RNA was used for cDNA synthesis in a reaction containing 0.3 μl of random primer, 1 μl of 10 mM dNTP, 2 μl of 0.1 M DTT and 200 U of M-MLV reverse transcriptase (Invitrogen). Primers used for *SyNOS* were: forward 5′tcggcagcaccgtctatgaa3′and reverse 5′tcggcttgtcctttgatttcg3′, and for the RNase P RNA Gene *RNPB* primers were: forward 5′cggctcaaagcaaggctcaa3′ and reverse 5′gatgcgattacggacgactgc3′.

### NO production and pigment content in Synechococcus PCC 7335 cultures

*Synechococcus* PCC 7335 cultures were grown in Erlenmeyer flasks containing ASNIII medium 17 at 25 °C under a 12 h:12 h (light:dark) photoperiod. NO content in *Synechococcus* and *E. coli* cultures was measured using the NO fluorescence probe DAF-FM DA (Invitrogen). DAF-FM DA (10 μM) was added to the *Synechococcus* PCC 7335 culture 40 min before measurement. NO production was initiated by the addition of the substrate L-Arginine with or without L-NAME. NO fluorescence intensity (excitation 495 nm; emission 515 nm) was measured using a fluorescence plate reader (Fluoroskan Ascent; Thermo Electron). Phycoerytrin (PE) concentration was determined spectroscopically at 542 nm with a ε for the hexamer of 2.15 × 10^6^ cm^−1^ M^−1 ^^64^, and chlorophyll concentration by absorption at 663 nm from acetone 80% extracts with ε 82 mM^−1^ cm^−1 ^^65^.

### Statistical analyses

Statistical analyses were conducted with SigmaPlot v. 11.0 software (Systat Software Inc., CA, USA). The normality of the data was tested Shapiro–Wilk test. Statistical significance was determined by Student’s t test for pairwise comparisons when the assumptions of normality and/or homogeneity of variances were not violated. Mann–Whitney test was performed when the assumptions of normality and/or homogeneity of variances were violated. One way ANOVA was used for multiple comparisons with *post hoc* comparisons by the Holm-Sidak multiple comparison test.

## Electronic supplementary material

Supplementary Information

Supplementary Dataset

## Data Availability

The materials, protocols and dataset generated and/or analyzed during the current study are available from the corresponding author upon reasonable request.
