# Supplementary material for: A singular nitric oxide synthase with a globin domain found in Synechococcus PCC 7335 mobilizes N from arginine to nitrate
Source: Sci Rep. 2018 Aug 21;8:12505. doi: 10.1038/s41598-018-30889-6 (PMC6104048; doi:10.1038/s41598-018-30889-6)
Supplement: Supplementary file 1 — Supplementary Information [file 41598_2018_30889_MOESM1_ESM.pdf]

Supplementary information

A singular nitric oxide synthase with a globin domain found in *Synechococcus PCC 7335* mobilizes N from arginine to nitrate

Natalia Correa-Aragunde, Noelia Foresi, Fiorella Del Castello, Lorenzo Lamattina.

Instituto de Investigaciones Biológicas. Facultad de Ciencias Exactas y Naturales, Universidad Nacional de Mar del Plata - CONICET, CC 1245, 7600 Mar del Plata, Argentina.

*Corresponding author: Dr. Lorenzo Lamattina, E-mail: lolama@mdp.edu.ar

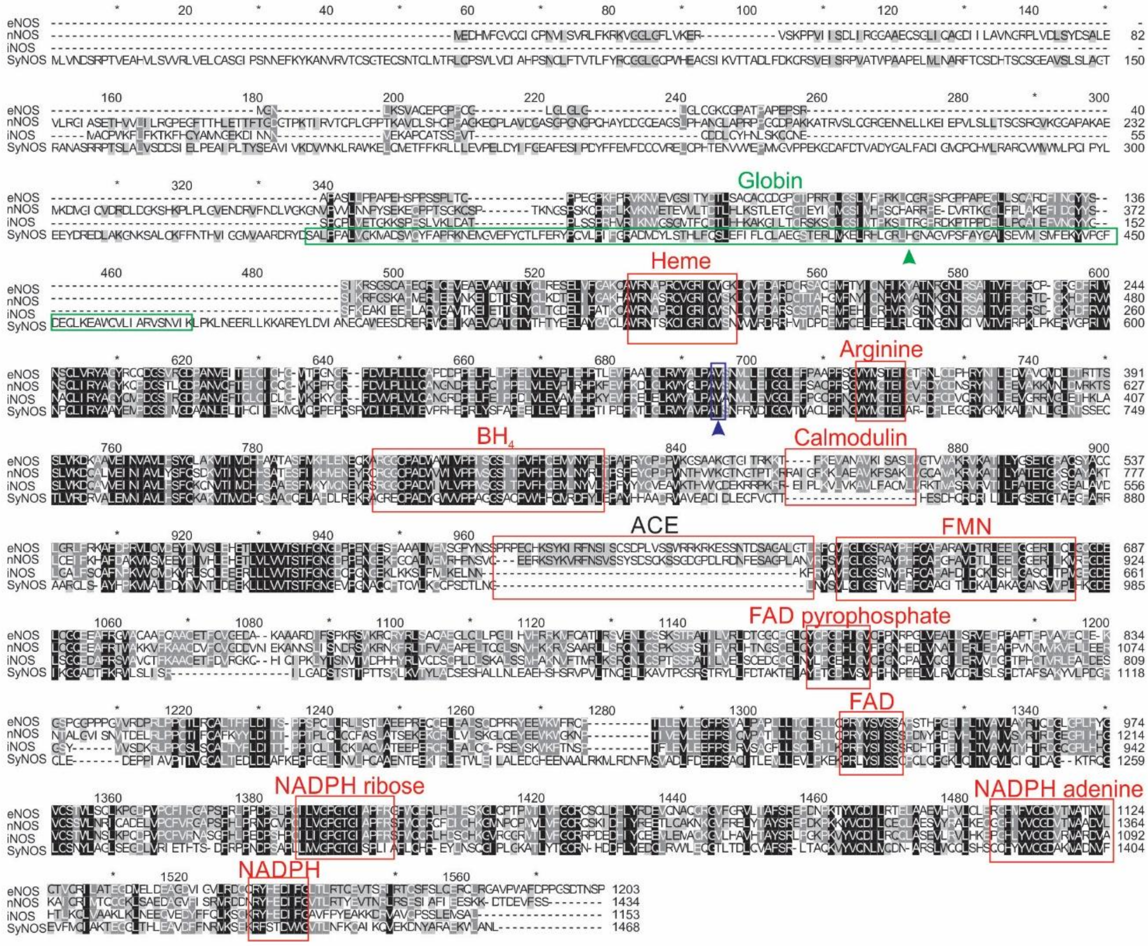

Supplementary Fig. S1. Alignment of *Synechococcus* PCC 7335 NOS (SyNOS) and human NOS sequences. The sequences of SyNOS and human eNOS, nNOS, and iNOS were aligned using ClustalX and Genedoc software. Black boxes indicate conserved residues in all four sequences, dark-gray boxes represent conserved residues in three sequences, and light-gray boxes represent conserved residues in two sequences. Amino acids that share no similarity are unshaded. Putative substrate binding site for Arginine (Arg) and cofactor binding sites for zinc, Heme, BH_4, CaM, FMN, FAD pyrophosphate, FAD isoalloxazine, NADPH ribose, NADPH adenine, and the C-terminal domain of NADPH are shown. The blue arrowhead indicates the change from Val to Ile in SyNOS. The globin domain in SyNOS sequence is depicted as a green box. The green arrowhead indicates the His residue that coordinates the Heme group.

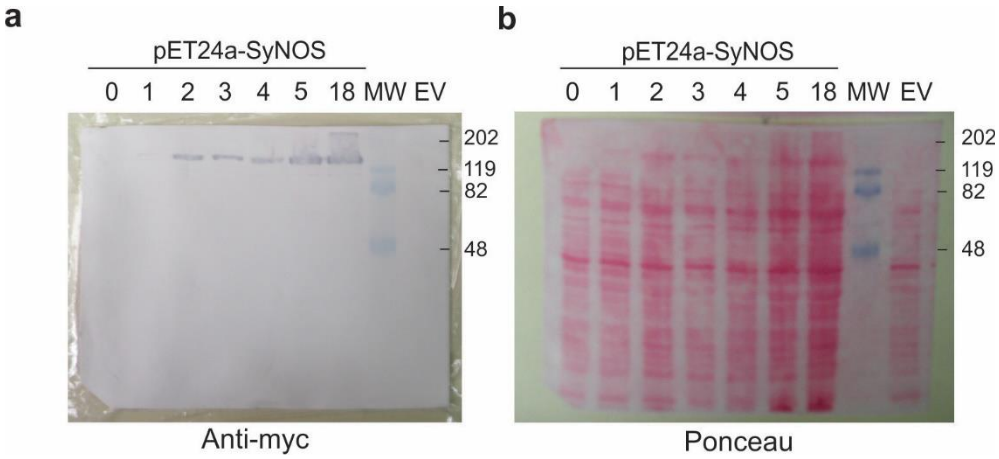

Supplementary Fig. S2. Expression of recombinant NOS from *Synechococcus* PCC 7335 in *E. coli*. The open reading frame of SyNOS was cloned into pET24a vector with a c-myc tag in the C terminus (pET24a-SyNOS). Empty vector (EV). The expression of SyNOS was induced by addition of 0.1 mM IPTG for different time points. (**a**) Immunoblot showing the expression of SyNOS revealed with anti-myc antibody. (**b**) Ponceau staining. Molecular weight standards (MW) are shown on the right side of each panel in kDa.

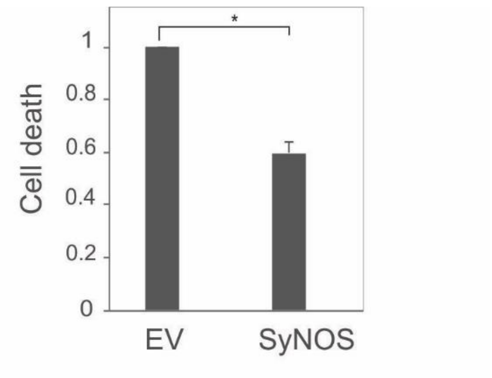

Supplementary Fig. S3. Cell death of *E. coli* cultures expressing SyNOS or EV induced by IPTG 0.1 mM for 4 h. The viability was assayed with Sytox green fluorescent probe and measured in a fluorometer. Error bars represent ± SE (n = 3). Asterisks represent statistically differences compared to bacteria expressing the EV (t-test, $P < 0.05$).

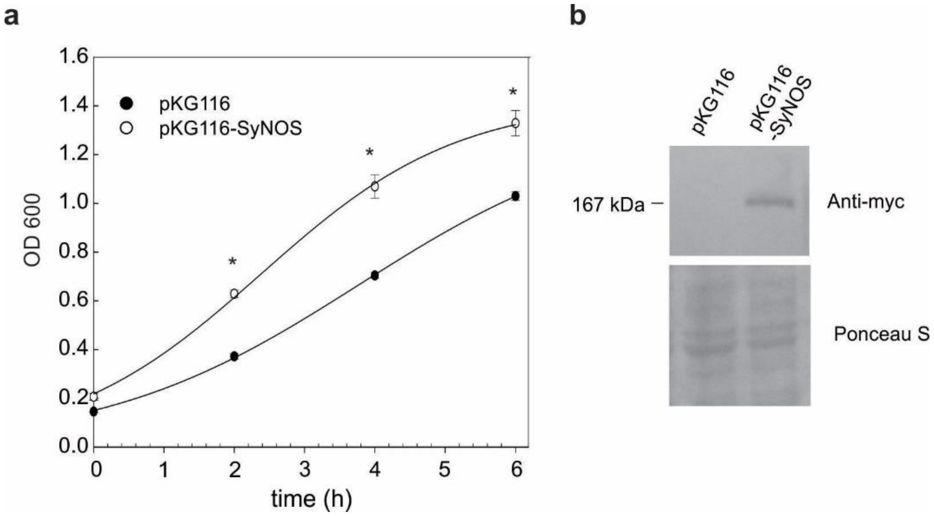

Supplementary Fig. S4. Expression of recombinant SyNOS in the wild type *E. coli* K12-derived strain *RP437* and its effect on the growth rate. (**a**) Growth curves of *E. coli RP437* cultures growing in LB media expressing recombinant SyNOS or the empty vector pKG116 induced by 0.45 µM salicylic acid (SA). Growth was followed by measuring OD 600. Data represent three independent experiments. Asterisks denote statistical difference compared to E. coli expressing the empty vector pKG116. (**b**) immunoblot showing the expression of SyNOS at 6 h of SA induction.

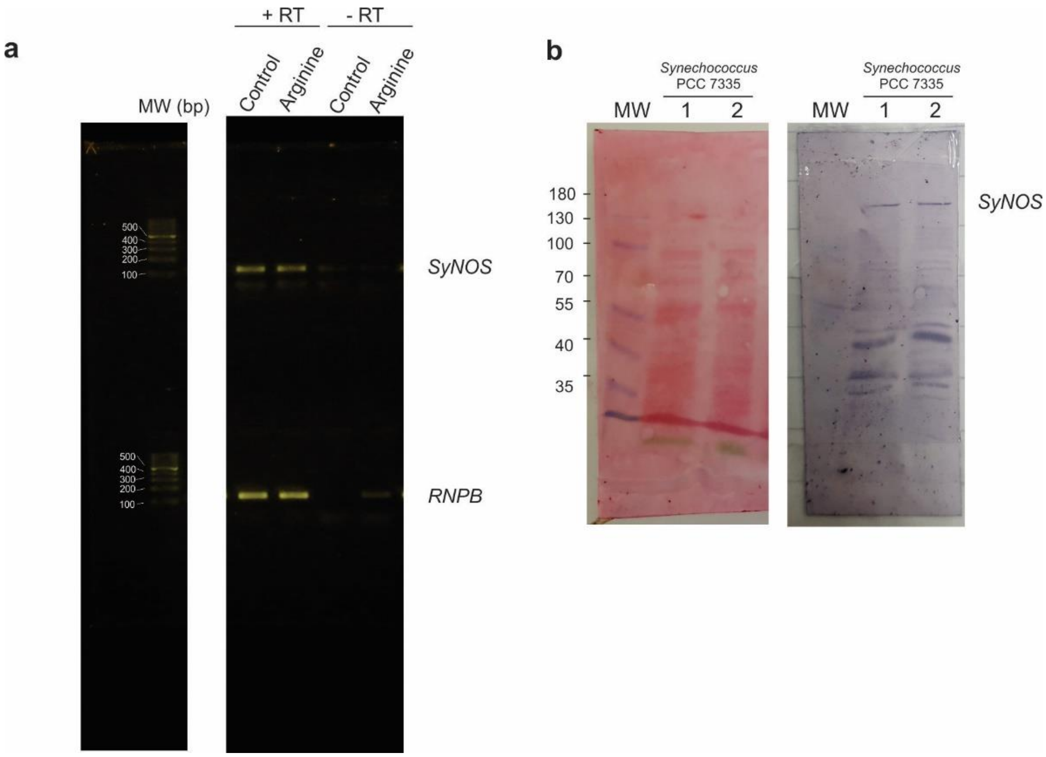

Supplementary Fig. S5. Analysis of full length blots of RT-PCR and western blot showing the expression of SyNOS in *Synechococcus* PCC 7335. (**a**) RT-PCR analysis showing the expression of SyNOS in *Synechococcus* PCC 7335. The RNase P RNA (RNPB) was used as a control for cDNA loading. MW: molecular weight standard in base pair. (**b**) Detection of SyNOS protein in

Synechococcus PCC 7335. Synechococcus protein extract was loaded in lines 1 and 2. SyNOS protein was detected with a specific antibody (Genscript). MW: Molecular weight standards in kDa.

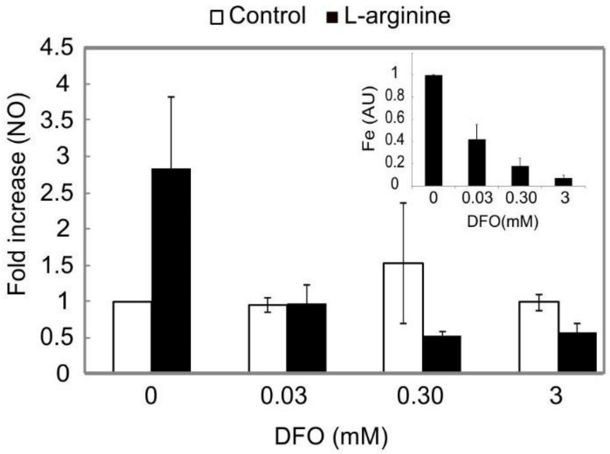

Supplementary Fig. S6. Requirement of Fe for the arginine-dependent increased in NO production in *Synechococcus* PCC 7335. Synechococcus cultures were treated with different concentrations of the iron chelating agent deferoxamine (DFO) for 45 min. NO production was measured with the fluorescence probe DAF-FM DA with or without the addition of 5 mM arginine. Inset, iron content after DFO treatment. Iron was measured with the fluorescent probe calceine.

Supplementary Table S1. Accession numbers of NOS sequences used for the phylogenetic analysis.

Accession number	Species
AAF25682	*Drosophila melanogaster*
XP 001660328	*Aedes aegypti*
BAH14964	*Gryllus bimaculatus*
NP 001012980	*Apis mellifera*
NP 851380	*Bos taurus*
CAA53950	*Homo sapiens*
NP 068610	*Rattus norvegicus*
ABO21653	*Physarum polycephalum*
XP 001421937	*Ostreococcus lucimarinus*
XP 003083764	*Ostreococcus tauri*
CCO66498	*Bathycoccus prasinos*
ISIM2037077	*Nephroselmis pyriformis*
DRGY2007432	*Chaetosphaeridium globosum*
KST62223	*Mastigocoleus testarum*
KJH70221	*Aliterella atlantica*
WP 019497077	*Calotrix sp. PCC 7103*
YP 006995309	*Anabaena sp. 90*
KHG40833	*Aphanizomenon flos-aquae*
YP 603740.1	*Deinococcus geothermalis*
ETK27041	*Paenibacillus larvae*
WP 006671088	*Halobiforma nitraredurens*
YP 326610	*Natronomonas pharaonis*
YP 006790088	*Exiguobacterium antarcticum*
YP 004093749	*Bacillus cellulosilyticus*
YP 007401967	*Geobacillus sp. GHH01*
AHL70582.1	*Bacillus pumilus*
WP 006637417	*Bacillus sonorensis*
WP 019257766	*Bacillus subtilis*
WP 018825545	*Salinispora arenícola*
YP 003391026	*Spirosoma linguale*
YP 007143319	*Crinalium epipsammum*
WP 006458277	*Synechococcus sp. PCC 7335*
YP 007051528	*Nostoc sp. PCC 7107*
EJK 55330	*Thalassiosira oceánica*
WP 017655351	*Fortiea contorta*
WP 010290958	*Kurthia massiliensis*
